# QT Dispersion and T Wave Peak–to–end Interval Dispersion in Children with Kawasaki Disease

**Published:** 2013-09-01

**Authors:** Hamid Amoozgar, Maryam Ahmadipour, Anis Amirhakimi

**Affiliations:** 1 Cardiovascular Research Center, Shiraz University of Medical Sciences, Shiraz, IR Iran; 2 Division of Pediatric Cardiology, Department of Pediatric, Shiraz University of Medical Sciences, Shiraz, IR Iran

**Keywords:** Kawasaki, Dispersion, Electrocardiogram

## Abstract

**Background:**

The main complication of Kawasaki disease is the Coronary Artery (CA) involvement and long term follow up of patients depends on the severity of coronary arterial aneurysms, ischemia, and thrombosis. Early diagnosis of these complications can lead to a more desirable outcome for patients. Myocardial ischemia can prolong QT dispersion and increase the risk of cardiac arrhythmias as well as sudden cardiac arrests. Also, T wave peak–to–end (Tp-Te) interval dispersion, which provides a valuable index of transmural dispersion of repolarization, can trigger the arrhythmia.

**Materials and Methods:**

We evaluated the non-corrected QT interval dispersion (QTD) and the corrected QT (QTc) dispersion and measured Tp–Te interval dispersion in 49 Iranian children (28 males and 21 females) with the diagnosis of Kawasaki disease (KD) in the acute phase and 49 age-matched controls in a prospective study from 2009 to 2012. Student’s t-test and Pearson correlation were used to analyze the data. All the statistical analyses were performed through the SPSS 16. Besides, P<0.05 was considered as statistically significant.

**Results:**

Patients with KD had significantly longer QTc dispersion (0.099±0.055 s versus. 0.040±0.018 s; P<0.001), non-corrected QT dispersion (0.075±0.046 versus 0.042±0.019; P<0.001), and Tp-Te dispersion (0.047±0.054 versus 0.022±0.011; P=0.015). The patients with elevation in white blood cell count (above 15000) had a statistically significant increased in QTD (P=0.011). No significant correlation was found between coronary involvement and repolarization indexes.

**Conclusions:**

In conclusion, the QT interval (corrected or non-corrected) and the Tp-Te dispersion significantly increased in the patients with KD which shows repolarization changes during the acute phase of KD. However, there is no correlation between the QT interval and the coronary involvement.

## 1. Background

Kawasaki Disease (KD) is an inflammatory condition with frequent involvement of coronary arteries (1). Occurrence of necrosis, tissue death, fibrosis, and proliferation of cells, which are associated with inflammation in the inner layer of the vascular wall, showed that KD is a necrotizing vasculitis (2). Cardiac complications are the most significant aspect of the disease. In addition, KD causes vasculitic changes in the CA due to the inflammation of the blood vessels and the subsequent CA aneurysm which is associated with thrombosis, stenosis, and myocardial infarction (3, 4). About 10-18% of the patients with KD present CA aneurysms with much higher prevalence among the children who are not treated early in the course of the disease (2, 5, 6). Overall, about 2% of the patients die from the complications of coronary vasculitis. With early standard treatment of KD, the acute symptoms of the disease tend to improve and, at the same time, the probability of CA aneurysm decreases significantly (6, 7). Early diagnosis of myocardial ischemia will lead to more desirable outcomes as well as treatments for these patients (3, 8). Several studies reported that myocardial damages result in altered electric potential distributions which are manifested through the increased QT dispersion (7, 8). Ischemic myocardial failure results in the instability of myocardial repolarization as well as electrophysiological changes which occur due to the inflammation which may create arrhythmia (9). The dispersion of repolarization is due to differences in the action potential times in myocardial cells (10). Transmural dispersion of repolarization in the heart has been linked to a variety of arrhythmic manifestations. Three electrophysiological cell types have been identified in the ventricular myocardium: endocardial, epicardial, and M cells. Differences in the period of repolarization of these three types of cells result in the reflection of the T-wave in the surface ECG (11). The peak of the T-wave was shown to coincide with the epicardial repolarization and the end of the T-wave with the depolarization of the M cells; therefore, T peak–T end provides a measure of the transmural dispersion of the repolarization and may provide a helpful index for the detection of life-threatening arrhythmias (12). As a result, detection of repolarization changes could be useful for early diagnosis of carditis and ischemia in KD patients. In this study, we evaluated the QT and the Tp-Te interval dispersion changes in the patients with KD as well as their correlation with other risk factors of the coronary involvement.

## 2. Materials and Methods

Between April 2009 and April 2012, we consecutively enrolled 49 children with the diagnosis of KD, according to the definition by the American heart association, who were in the acute phase of the disease (13). Forty nine normal children without any history of cardiac diseases who had referred to the pediatric clinic for normal children physical exam were also recruited as the control group. The QT analysis was done on a surface 12-lead electrocardiogram (25 mm/s paper speed, 10 mv/1 mm). All electrocardiograms were recorded with the participants in the supine position. Recordings were made using a digital electrocardiogram machine (Alicia Diagnostics, Sanford, FL, USA). The digitally recorded electrocardiogram tracings were evaluated by a digital clipper in Corel photo paint software version 13 (Ottawa, Canada). Magnification of the electrocardiogram made a fine determination of the measurement points. The QT interval was measured from the beginning of the QRS complex to the end of the T wave. If the U wave was less than 50% of the T wave, it was excluded from the QT. The QT dispersion was determined as the difference between the maximum and the minimum QT intervals in the 12 lead electrocardiogram of each patients. In order to measure the Tp-Te, the QT interval and the Q to T peak interval were calculated in precordial leads and their difference was also computed. The difference between the longest and the shortest Tp–Te intervals in each patient was considered as the T peak to T end dispersion. In patient with coronary aneurysm, exercise test was performed 6 month after the acute phase and if any sign of ischemia was detected, angiography was performed. The statistical analyses were performed using the SPSS statistical software, version 16 (SPSS, Inc. Chicago, IL). The data were expressed as mean±one standard deviation. Student’s t test was used to compare the mean values of the patients and the control group with the probability values being statistically significant at 0.05 levels. Besides, Pearson correlation was used to evaluate the relationship between the parameters.

## 3. Results

Forty nine patients (28 males and 21 females; range: 1-10 years; mean age: 4.12±2.67 years) with a history of KD in the acute phase were evaluated. Ten patients had the coronary artery aneurysm and 3 cases had developed a giant aneurysm (>8mm). Means of QT in the patients and controls are shown in Table 1. The non-corrected QT dispersion in the patients with KD was 0.075±0.046 s versus 0.042±0.019 s in the controls (P<0.001).

**Table 1.** Mean QT in the Patients with Kawasaki Disease and Controls (mean±SD)

QT	Patients with KD (mSec)	Normal Subject(mSec)	*P value*
I	0.309±0.078	0.341±0.041	0.035
II	0.320±0.066	0.345±0.040	0.041
III	0.317±0.074	0.339±0.047	0.263
avF	0.320±0.071	0.341±0.039	0.139
avL	0.312±0.069	0.338±0.044	0.433
avR	0.320±0.069	0.323±0.042	0.173
V1	0.330±0.064	0.341±0.041	0.398
V2	0.326±0.081	0.350±0.045	0.144
V3	0.319±0.069	0.347±0.043	0.038
V4	0.326±0.073	0.346±0.041	0.167
V5	0.331±0.075	0.344±0.040	0.338
V6	0.327±0.066	0.338±0.040	0.394

The mean corrected QT in the 12 leads of the patients and the controls are presented in Table 2. The QTc dispersion in the patients with KD was 0.099±0.055 s compared to 0.040±0.018 s in the controls (P<0.001).

**Table 2.** Mean Corrected QT in the Patients with Kawasaki Disease and Controls (mean±SD)

QT	Patients with KD (Second)	Normal Subjects(Second)	*P value*
I	0.390±0.062	0.325±0.037	<0.001
II	0.410±0.025	0.330±0.039	0.0001
III	0.404±0.040	0.322±0.040	0.0001
avF	0.408±0.033	0.325±0.030	0.0001
avR	0.409±0.030	0.323±0.040	0.0001
avL	0.399±0.039	0.308±0.03	0.0001
V1	0.424±0.033	0.325±0.037	0.0001
V2	0.416±0.059	0.334±0.04	0.0001
V3	0.408±0.034	0.331±0.030	0.0001
V4	0.414±0.034	0.330±0.04	0.0001
V5	0.421±0.036	0.329±0.03	0.0001
V6	0.419±0.036	0.32 1±0.04	0.0001

The mean Tp-Te interval in the 12 leads electrocardiograms of the patients and controls are shown in Table 3. The Tp-Te interval dispersion in the patients and the control group was 0.047±0.054 s and 0.022±0.011 s, respectively ( *P *=0.01).

**Table 3.** Mean T Peak to T End in the Patients with Kawasaki Disease and the Controls (mean±SD)

T peak-to-end	Patients with KD (Sec)	Normal Subjects(Sec)	*P value*
V1	0.059±0.017	0.072±0.012	0.000
V2	0.062±0.058	0.074±0.018	0.273
V3	0.060±0.020	0.072±0.015	0.004
V4	0.069±0.024	0.076±0.017	0.139
V5	0.066±0.024	0.074±0.010	0.026
V6	0.061±0.018	0.073±0.011	0.002

The high risk patients with age <5 years versus >5 y, prolonged fever (>5days), platelet count >450000, duration of the disease >10 days, coronary artery aneurysm, giant aneurysm, polymorphous exanthema, bilateral conjunctival injection, changes in lips, cervical lymphadenopathy, and edema in extremities did not reveal any significant increase in the QTD (P>0.050). Moreover, no statistically significant correlation was found between lymphocyte count, platelet count, serum C reactive protein, and duration of disease and repolarization changes (P>0.05). The patients with elevation in white blood cell count above 15000 had a significantly more prolonged QTD (P=0.011).

Furthermore, no significant difference was observed between the 10 patients with coronary aneurysm and other patients regarding QT dispersion, QTC dispersion, and Tp-Te dispersion (0.073±0.017 vs. 0.078±0.056, P=0.783; 0.090±0.025 vs. 0.105±0.079, P=0.550; 0.048±0.016 vs. 0.039±0.018, P=0.174).

## 4. Discussion

Involvement of coronary arteries, which may be the leading cause of aneurysm formation, is the most important complication of KD which may lead to flow stasis and thrombosis. Myocardial ischemia and infarction are serious sequels in pediatric patients.

For early diagnosis of myocardial ischemia, some studies reported altered repolarization parameters, such as ventricular late potential and QTD, to result in dangerous ventricular arrhythmias. The exact mechanism of the increased QTD in KD is not determined. One possibility is that the QTD may be affected by the extent of the myocardial damage due to ischemia (14, 15).

In this study, no statistically significant association was found between the QTD and asymptomatic coronary aneurysm, male gender, older age, prolonged fever, high platelet count, and high white blood cell count in the patients with KD.

In a study, Dahdah et al. showed that electrocardiogrphic depolarization and repolarization would be prolonged after KD. They also found a significantly longer dispersion in the patients with advanced coronary artery lesions. Moreover, they suggested a prospective electrocardiography and QTD analysis evaluation during the convalescent phase up to one year after KD in the patients who had a significant cardiac involvement (14).

Osada et al. also reported the same findings in their patients, which is in line with our results. However, they reported that the increase of QTD could not certainly indicate the progression of the myocardial ischemia after KD (16).

Moreover, Ogawa et al. showed a high incidence of late potentials in the KD patients with myocardial ischemia (17).

In a study which was performed by Higham et al., local changes in action potential, conduction pathway, and neurohumral factors due to the myocardial ischemia were believed to increase the QTD (18).

The increased QT interval is due to the prolongation of the action potentials and is considered as a marker of the dispersion of the ventricular repolarization. Several studies revealed that the QTD could predict ventricular arrhytmogenicity. Furthermore, Yamaguchi et al. demonstrated that the QTD in the adult patients with Torsades De Pointes (TDP) was significantly higher in comparison to those without TDP (19).

Nevertheless, limited data are available on the issue. The present study revealed that the QTD was significantly higher in the patients with KD compared to the control group, which might be due to the carditis.

Also, recent studies showed that the second part of the T wave demonstrated the arryhtmogenic background (18, 19), and the Tp-Te interval of the T wave might be a helpful predictor of arrhythmia and sudden cardiac events. Furthermore, the dispersion of the terminal part of the T wave is a more appropriate marker of transmural dispersion compared to the QT dispersion. However, this issue was not proved in pediatric patients. In fact, little investigation of the issue has been conducted on children. In the present study, we demonstrated a higher Tp-Te dispersion in KD which is related to the transmural dispersion of the repolarization in inflamed myocardial cells of these patients. However, none of our patients had ischemic changes or arrhythmia in their surface electrocardiogram. Besides, no significant correlation was found between the ischemic changes and other parameters of inflammation. Our study revealed statistically significant depolarization abnormalities. Although a significant number of the patients under study had the coronary involvement diagnosed in echocardiography, none of these patients developed ischemic changes in follow up exercise tests and coronary angiography 3 months after their disease. Similarly, Charles et al. came to the conclusion that the increased dispersion of the ventricular repolarization was not correlated with CA abnormalities in KD (20).

Our study showed that in the acute phase of KD, QT dispersion, QTC dispersion, and Tp-Te dispersion increased due to the inflammatory process of KD or carditis, but could not predict the development of coronary aneurysm.

In conclusion, the QT interval (corrected or non-corrected) and the Tp-Te dispersion increased significantly in the patients during the acute phase of KD, which shows repolarization changes. However, no statistically significant correlation was observed between the repolarization changes and the coronary aneurysm formation.

**Limitations**

Diagnosis of coronary anurysem in this study was according to two dimensional echocardiography. Although angiography is the gold standard for detection of coronary aneurysm and obstruction, it is not recommended in the acute phase of KD due to its invasiveness in this phase. However, some studies have shown fine sensitivity and specificity of echocardiography in detection of coronary anurysem due to higher proximal coronary involvement in KD (21).
